# Living longer but in poor health: healthcare system responses to ageing populations in industrialised countries based on the Findings from the Global Burden of Disease Study 2019

**DOI:** 10.1186/s12889-024-18049-0

**Published:** 2024-02-22

**Authors:** Minjae Choi, Joshua Kirabo Sempungu, Eun Hae Lee, Yo Han Lee

**Affiliations:** 1 Institute for Future Public Health, Graduate School of Public Health, Korea University, Seoul, Republic of Korea; 2 Department of Preventive Medicine, Korea University College of Medicine, 73, Goryeodae-ro, Seongbuk-gu, Seoul, Republic of Korea; 3 Program in Public Health, Graduate School, Korea University, Seoul, Republic of Korea

**Keywords:** Life expectancy, Health-adjusted life expectancy, Healthcare system, Healthcare burden, Ageing population

## Abstract

**Objectives:**

This study aimed to examine changes in life expectancy (LE), health-adjusted life expectancy (HALE), unhealthy years of life, and disease burden of older people in industrialised countries and associations with health systems.

**Methods:**

We used estimates of LE and HALE, unhealthy years of life, years of life loss (YLL), years lived with disability (YLD) for individuals aged 70 years and over in 33 industrialised countries from 1990 to 2019 from the Global Burden of Disease Study 2019. A linear regression analysis was conducted to examine the association of health outcomes with the Healthcare Access and Quality (HAQ) index.

**Results:**

LE and HALE increased with improved HAQ index from 1990 to 2019. However, the number of unhealthy years of life increased. An increased HAQ index was associated with decreases in YLL. However, changes in YLD were relatively small and were not correlated with HAQ index.

**Conclusions:**

The healthcare system needs to more address the increased morbidity burden among older people. It should be designed to handle to healthcare needs of the ageing population.

**Supplementary Information:**

The online version contains supplementary material available at 10.1186/s12889-024-18049-0.

## Background

Life expectancy has steadily increased in most countries for last few decades [1]. Particularly in high-income countries, the majority of newborns are expected to reach their 80s and even beyond [2]. These industrialised countries show a high likelihood that life expectancy will continue to rise by 2030, driven by enhanced longevity in the older population [2]. For example, females in South Korea have a 57% probability of living to 90 years or beyond [2]. Moreover, epidemiological studies have demonstrated a rapid population ageing trend, with individuals aged over 70 years constituting the fastest-growing demographic in high-income countries [3]. This trend suggests that a greater number of older adults will lead longer lives in these countries.

However, an increased life expectancy for older adults does not necessarily imply additional healthy years, although an extended lifespan is a notable achievement [4]. Health-adjusted life expectancy (HALE), which defines the average number of healthy years a person of a specific age can expect to live, has been proposed as a health indicator [5]. Monitoring trends in both life expectancy and HALE serves to assess the population health [6]. Understanding the extent of the difference between life expectancy and HALE is crucial to achieving healthy ageing, suggesting that both measures increase with minimal differences between them [7].

There has been a long-standing debate about the difference between life expectancy and HALE. Some scenarios have been proposed to understand the healthcare burden [8, 9]. One scenario suggests that as life expectancy increases while the years lived with underlying illness or disability decrease, referring to “compression of morbidity” [10]. Conversely, another scenario, termed “expansion of morbidity”, suggests that as life expectancy rises, the years of good health decrease [11]. Understanding whether older adults live longer or shorter lives in poor health is important, as these scenarios imply different patterns of disease burden and provide public health policy implications in a given country for planning healthcare strategies to meet the needs of the ageing population [12].

Healthcare for the ageing population has become essential as it helps maintain their health and promotes active engagement of older people in society [3]. Accordingly, healthcare systems should be designed to provide access to high-quality healthcare for the ageing population [13] and be prepared for a healthy ageing society where older people may have one or more chronic health conditions, but these conditions have little influence on their wellbeing because they are well controlled [14]. Hence, evaluating whether healthcare systems address healthcare needs and responses to the aging population is imperative [15]. Despite its importance, cross-national studies on life expectancy and HALE in older adults and their correlation with healthcare performance are limited [3, 16] Most studies focused on individual national populations [17–21]. Furthermore, the varying definition of healthcare system performance and related indicators among different countries poses challenges in comparing these metrics within a single study [22]. The Global Burden of Disease Study (GBD) 2019 provides data to investigate trends in population health, including life expectancy, HALE, and disease burden [23]. Moreover, the GBD 2019 introduced Healthcare Access and Quality (HAQ) index, which enable to assess whether health systems provide access to quality health care for all ages using coherent estimation methods across countries and regions [15]. To our knowledge, only one study investigated the trend of healthcare needs among elders and how changes in HAQ in low-income countries (LICs) [24]. However, there was limited research for high-income countries.

This study aimed to investigate changes in life expectancy, HALE, and disease burden among individuals aged 70 years or above from 1990 to 2019 in industrialised countries. Additionally, we examined the association of healthcare performance with life expectancy, HALE, and disease burden to assess whether the healthcare system meets the needs of the ageing population in industrialised countries.

## Methods

### Overview of the GBD 2019

We used estimates from the GBD 2019, which provides estimates for prevalence, incidence, mortality, years of life lost (YLL), years lived with disability (YLD), and disability-adjusted life years (DALY) of 369 diseases and injuries, as well as life expectancy and HALE by sex, age group, and region from 1990 to 2019 [23]. Disease and injury causes were aggregated into a four-level hierarchy, with each level consisting of mutually exclusive causes. Level 1 causes comprise three broad groups: communicable, maternal, neonatal and nutritional diseases (CMNNs), non-communicable diseases (NCDs), and injuries. There are 22 causes in Level 2, 174 causes in Level 3, and 301 causes in Level 4 [23]. In the GBD 2019, 204 countries and territories were included and grouped into 21 regions [25]. More details on the analytic framework, methods, and data sources used to derive each measure in the GBD 2019 are described in previous publications [23, 25]. Briefly, the GBD study used all available data sources, assessed quality to reduce biases in each source, applied coherent statistical modelling methods, and generated estimates with 95% uncertainty intervals (UIs) [23]. Moreover, the GBD 2019 adhered to the guidelines for accurate and transparent health estimates reporting (GATHER) [23, 25].

### Data processing and country selection

We used estimates of life expectancy and HALE of those aged 70–74 years, as well as YLL, YLD, and DALY for those aged 70 years and above. YLD was computed by multiplying prevalence estimates of specific disease by their respective disability weights to evaluate morbidity burden [23]. YLL was estimated by multiplying the number of deaths by the remaining life expectancy at the age of death, assessing premature mortality burden [23]. DALY was calculated as the sum of the YLD and YLL. HALE was estimated using the Sullivan method with data for age-specific mortality rates, prevalence, and disability weights [26]. All data by age group were downloaded from the Global Health Data Exchange [27].

We used the HAQ index to assess performance of the healthcare system in each country. Developed as part of the GBD study, the HAQ index is a composite measure assessing healthcare access and quality for those aged 0–74 years, allowing for comparability across regions. It is based on mortality-to-incidence ratios (MIRs) and risk-standardised death rates (RSDRs) for healthcare-sensitive diseases that could be effectively treated with proper healthcare services, such as neoplasms and cardiovascular disease [15]. Scores range from 0 (indicating the highest MIRs and RSDRs) to 100 (indicating the lowest MIRs and RSDRs). Additionally, the HAQ Index values were also calculated for three select age groups: young (0–14 years), working (15–64 years), and post-working (65–74 years) population [15]. This study specifically used the HAQ Index for the post-working age group (65–74 years). Data of HAQ was obtained from the Global Health Data Exchange [28].

We selected 33 high-income countries, all members of the Organization for Economic Co-operation and Development (OECD) in 2019, based on the availability data for life expectancy, HALE, DALY, YLL, YLD, and HAQ in both 1990 and 2019, along with a population exceeding one million across the study periods. The 33 countries included in this study are: Australia, Austria, Belgium, Canada, Chile, Czech Republic, Denmark, Estonia, Finland, France, Germany, Greece, Hungary, Ireland, Israel, Italy, Japan, Latvia, Lithuania, Mexico, Netherlands, New Zealand, Norway, Poland, Portugal, Slovak Republic, Slovenia, South Korea, Spain, Sweden, Switzerland, United Kingdom (UK), and United States of America (USA).

### Statistical analysis

We examined changes in life expectancy and HALE by describing life expectancy and HALE for those aged 70–74 years in the 33 countries in 1990 and 2019. We also calculated unhealthy years of life (life expectancy-HALE), and the proportion of unhealthy years of life on total life expectancy [(life expectancy-HALE)/ life expectancy] [18] for those aged 70–74 in 1990 and 2019. A linear regression analysis was conducted to investigate associations between life expectancy, HALE, unhealthy years of life, and HAQ in 1990 and 2019. Additionally, we investigated the association between changes in these health indicators and changes in HAQ. We also examined changes in DALY, YLD, and YLL, both in numbers and rates among individuals aged over 70 years between 1990 and 2019. Furthermore, we analysed associations between YLD, YLL, and HAQ for each year in 1990 and 2019, as well as correlations in changes among YLD, YLL, and HAQ from 1990 to 2019.

## Results

**Fig. 1:**
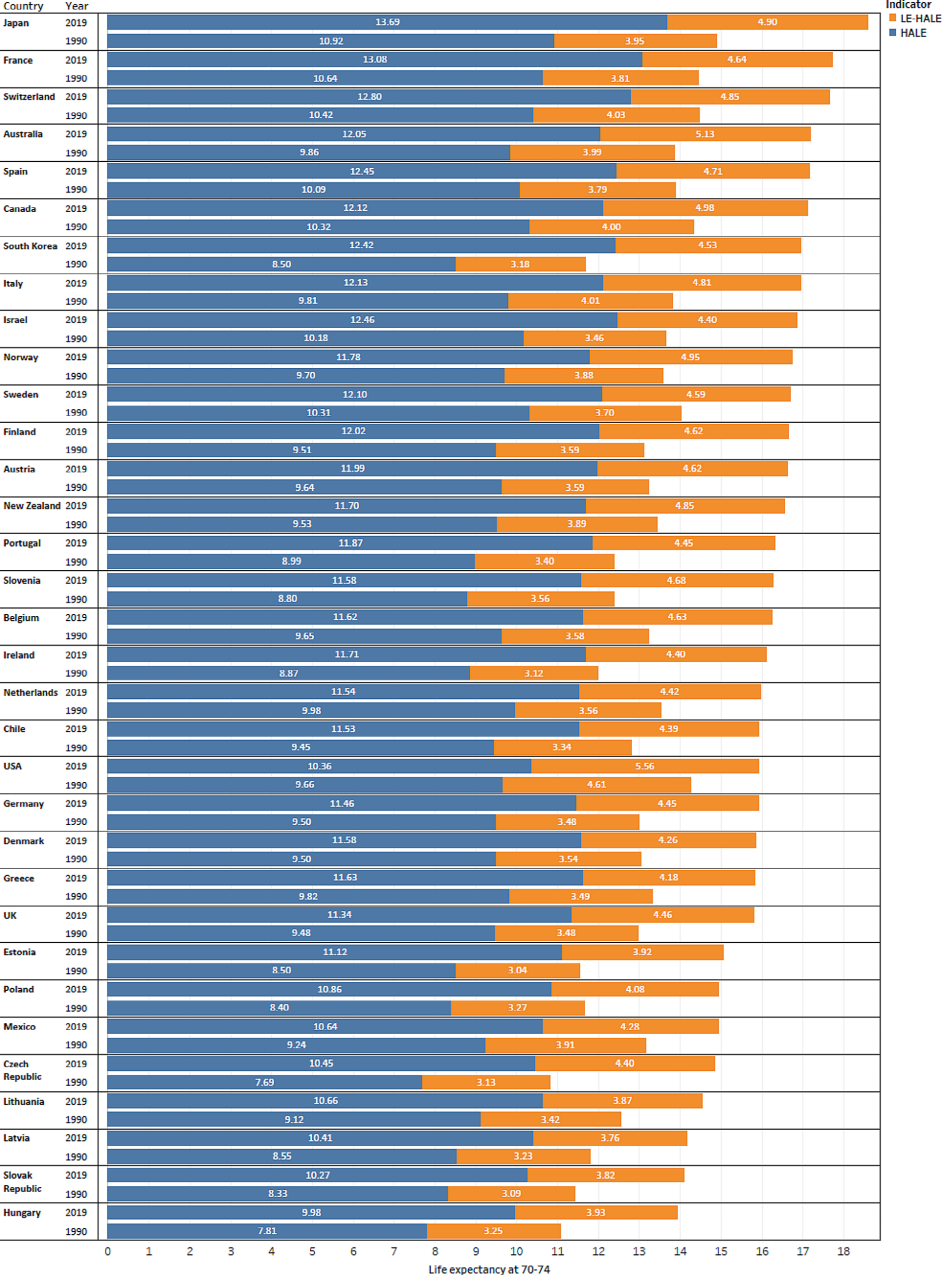
Life expectancy (LE), health-adjusted life expectancy (HALE), and unhealthy year (LE-HALE) at 70–74 years in 33 industrialized countries in 1990 and 2019

Life expectancy and HALE for those aged 70–74 years increased in all industrialised countries from 1990 to 2019 (Fig. 1). In 1990, the three countries with the highest life expectancy were Japan (14.9), Switzerland (14.4), and France (14.4), and the three countries with the lowest life expectancy were the Czech Republic (10.8), Hungary (11.1), and Slovak Republic (11.4). In 2019, the three countries with the highest life expectancy were Japan (18.6), France (17.7), and Switzerland (17.6), and the three countries with the lowest life expectancy were Hungary (13.9), Slovak Republic (14.1), and Latvia (14.2). Similarly, the country with the highest HALE in both 1990 and 2019 was Japan (10.9 in 1990, 13.7 in 2019), and the country with the lowest HALE was the Czech Republic (7.7) in 1990 and Hungary (10.0) in 2019. Regarding changes in life expectancy and HALE, the country with the highest increased life expectancy and HALE for three decades was South Korea (45.3% and 45.9%, respectively), USA (11.2% and 7.2%, respectively) was the country with the lowest increased life expectancy and HALE.

Although life expectancy and HALE increased, all industrialised countries showed increases of unhealthy years of life from 1990 to 2019. In 1990, the three countries with the highest unhealthy years of life were the USA (4.6), Switzerland (4.0), and Italy (4.0), and the three countries with the lowest unhealthy years were Estonia (3.0), Slovak Republic (3.1) and Ireland (3.1). In 2019, the three countries with the highest unhealthy years of life were the USA (5.6), Australia (5.1) and Canada (5.0), and the three countries with the lowest unhealthy years of life were Latvia (3.8), Slovak Republic (3.8), and Lithuania (3.9). The country with the highest increase in unhealthy years of life during the study period was South Korea (1.3 years, 40.6%), and the country with the lowest increase in unhealthy years of life was Mexico (0.4 years, 10.3%). The country with the highest proportion of unhealthy years of life in 1990 and 2019 was the USA (32.3% in 1990 and 34.9% in 2019), and the country with the lowest proportion of unhealthy years was Israel (25.4%) in 1990 and Estonia (26.1%) in 2019. There were fewer differences in the proportion of unhealthy years of life between 1990 and 2019 (See supplementary Table 1).

**Fig. 2:**
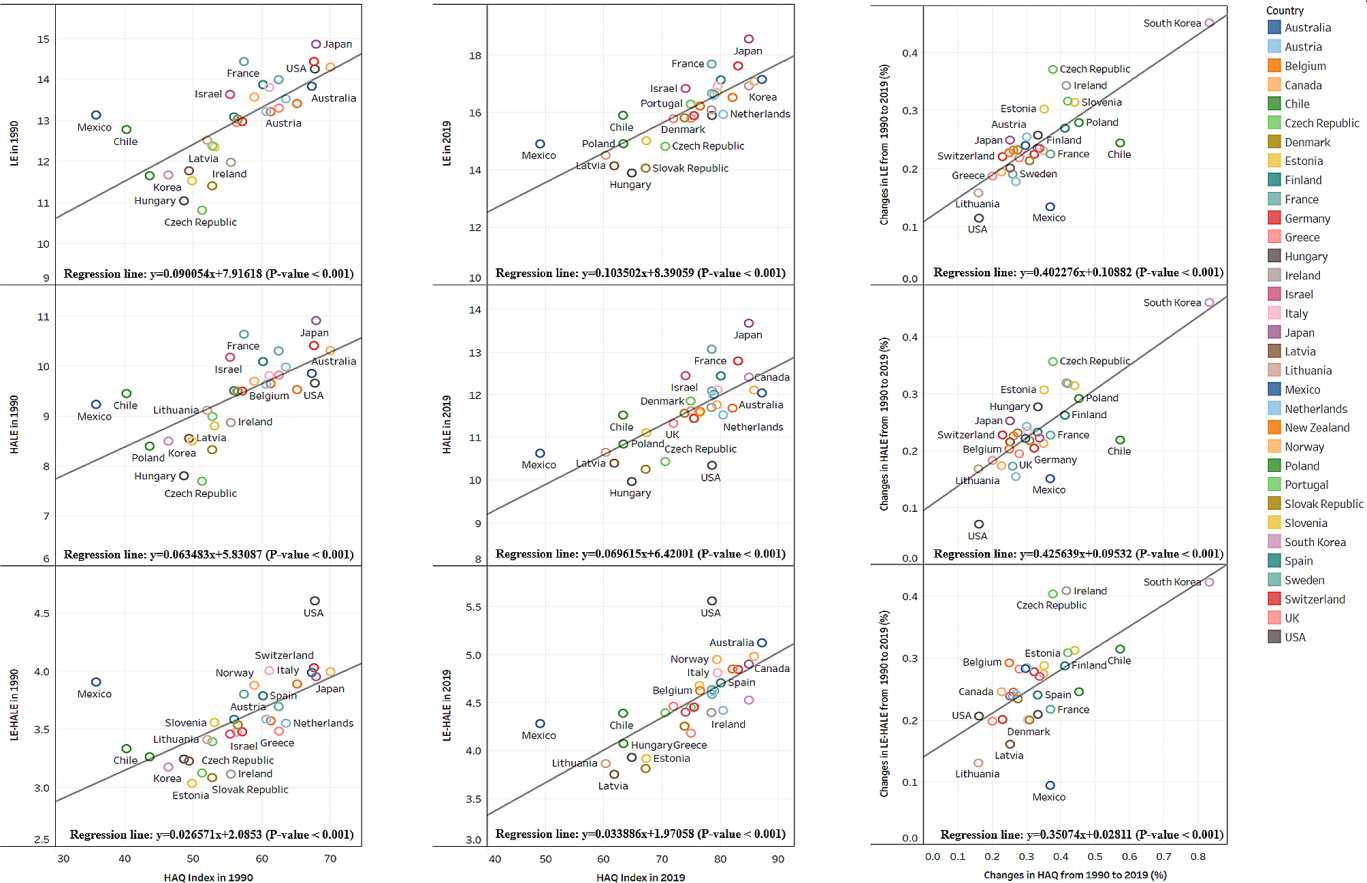
Association between life expectancy (LE), health-adjusted life expectancy (HALE), unhealth years (LE-HALE) at 70–74 years and HAQ Index from 1990 to 2019

We found significant associations between the HAQ index and each life expectancy and HALE for those aged 70–74 years in 1990 and 2019 (Fig. 2). Moreover, increases in HAQ were significantly associated with higher life expectancy and HALE. The association between unhealthy years of life and HAQ was also observed. In particular, South Korea experienced the most significant increase in HAQ with high increases in life expectancy and HALE but also showed the highest unhealthy years of life.

Table 1 shows DALY numbers and rates in 33 industrialised countries between 1990 and 2019 and the percent change from 1990 to 2019. All industrialised countries except Norway showed increased DALY numbers with varying percent changes from − 1.1 to 165.2%. Norway was the only country with reductions in DALY numbers from 545430.0 (95% UI 511679.0–582619.8) in 1990 to 539345.6 (95% UI 495094.4-589737.6) in 2019. However, all countries had reductions in DALY rates between 1990 and 2019. South Korea was the country that showed the highest decrease in DALY rates from 129165.4 (95% UI 122679.5–136199.9) in 1990 to 74713.6 (95% UI 68282.8–81757.7) in 2019 while the USA experienced the least decrease in DALY rates from 108304.3 (95% UI 100919.3–166446.4) in 1990 to 95798.4 (95% UI 88012.6–104067.7) in 2019.

**Table 1:** DALY numbers and rates per 100,000 people for adults 70 years and older in 33 industrialized countries between 1990 and 2019 and percent change from 1990 to 2019

Country	DALY numbers (95% UI)			DALY rates (95% UI)		
	1990	2019	Percent change (%)	1990	2019	Percent change (%)
Australia	1288134.1 (1206368.1–1377875.8)	2216097.9 (2021816.8–2425233.8)	72.0 (66.9 to 77.0)	106463.0 (99705.1–113880.1)	79180.9 (72239.2–86653.3)	−25.6 (−27.8 to −23.5)
Austria	893240.7 (840728.7–950505.8)	1029559.9 (946480.2–1123340.3)	15.3 (11.9 to 18.3)	116949.9 (110074.7–124447.5)	83691.1 (76937.7–91314.3)	−28.4 (−30.5 to −26.5)
Belgium	1117975.7 (1053745.6–1,188,763)	1383960.1 (1276941.1–1502207.7)	23.8 (20.4 to 27.1)	116223.0 (109545.7–123581.9)	88864.7 (81992.9–96457.4)	−23.5 (−25.6 to −21.5)
Canada	2036055.1 (1,903,407–2180849.1)	3586409.4 (3287725.6–3907701.5)	76.1 (72.1 to 80.3)	101234.4 (94639.0–108433.6)	79765.4 (73122.4–86911.3)	−21.2 (−23 to −19.4)
Chile	618190.5 (584874.1–657252.0)	1243459.7 (1146449.0–1351034.8)	101.1 (95.0 to 107.0)	115854.5 (109610.7–123175.0)	86235.7 (79507.9–93696.2)	−25.6 (−27.8 to −23.4)
Czech Republic	1241152.9 (1180902.7–1304974.8)	1421659.5 (1233209.1–1624636.7)	14.5 (2.0 to 28.3)	155846.4 (148281.1–163860.3)	96932.8 (84083.7–110772.3)	−37.8 (−44.6 to −30.3)
Denmark	658119.8 (619802.9–698637.7)	706944.5 (653011.4–763,341)	7.4 (4.3 to 10.5)	117584.0 (110738.1–124823.2)	86143.8 (79571.9–93,016)	−26.7 (−28.9 to −24.6)
Estonia	164774.3 (156581.8–173837.7)	177738.0 (151296.5–206020.8)	7.9 (−6.7 to 23.7)	138869.8 (131965.3–146508.3)	96415.3 (82071.9–111757.5)	−30.6 (−39.9 to −20.4)
Finland	530160.6 (498735.2–565247.4)	721009.8 (659,559–787624.2)	36.0 (31.1 to 40.6)	116000.4 (109124.4–123677.4)	81610.3 (74654.7–89150.3)	−29.6 (−32.2 to −27.3)
France	5348735.5 (5002163.1–5737377.4)	7159028.9 (6549804.5–7828901.5)	33.8 (30.3 to 37.0)	102491.7 (95850.7–109938.8)	75832.8 (69379.5–82928.5)	−26.0 (−28 to −24.2)
Germany	9732922.4 (9201324.3–10322332.7)	12026306.5 (11099031.6–13013426.3)	23.6 (20.5 to 26.8)	120467.3 (113887.6–127762.6)	90700.4 (83707.1–98145.1)	−24.7 (−26.6 to −22.8)
Greece	1049338.4 (988860.7–1115031.5)	1576759.8 (1465255.8–1693288.5)	50.3 (46.9 to 53.5)	110523.1 (104153.1–117442.3)	91826.6 (85332.9–98613.0)	−16.9 (−18.8 to −15.1)
Hungary	1269512.0 (1208272.5–1337644.8)	1412348.3 (1238533.7–1601204.0)	11.3 (−0.3 to 24.0)	149910.4 (142678.9–157955.9)	108228.1 (94908.7–122700.1)	−27.8 (−35.3 to −19.5)
Ireland	342550.8 (325482.7–361766.6)	404090.9 (372048.1–438929.2)	18.0 (13.5 to 22.5)	127520.3 (121166.4–134673.7)	83795.7 (77151.0–91020.0)	−34.3 (−36.8 to −31.8)
Israel	317,949 (299097.6–339320.1)	582415.2 (534387.8–633148.8)	83.2 (77.9 to 88.3)	104376.1 (98187.6–111391.8)	77746.1 (71,335–84518.5)	−25.5 (−27.6 to −23.4)
Italy	6012966.4 (5623266.4–6439360.7)	8513160.8 (7807182.8–9313120.1)	41.6 (38.6 to 44.5)	108556.9 (101521.4–116255.0)	82720.6 (75860.8–90493.6)	−23.8 (−25.4 to −22.2)
Japan	9085567.9 (8463529.5–9781967.8)	19271440.8 (17563950.0–21174411.9)	112.1 (107.1 to 117.0)	91909.7 (85617.2–98954.5)	69906.0 (63712.2–76808.9)	−23.9 (−25.7 to −22.2)
South Korea	1641621.5 (1559189.0–1731026.0)	3924525.2 (3586732.1–4294536.4)	139.1 (128.7 to 149.0)	129165.4 (122679.5–136199.9)	74713.6 (68282.8–81757.7)	−42.2 (−44.7 to −39.8)
Latvia	285263.4 (270737.2–301548.5)	297603.8 (262732.5–339062.9)	4.3 (−5.9 to 16.8)	137167.3 (130182.5–144,998)	105787.0 (93391.5–120524.2)	−22.9 (−30.4 to −13.7)
Lithuania	322661.9 (304997.0–342374.0)	409645.4 (357036.8–465966.2)	27.0 (13.3 to 42.1)	126594.3 (119663.6–134328.3)	102382.4 (89234.0–116458.7)	−19.1 (−27.9 to −9.5)
Mexico	2356507.3 (2206884.6–2522506.9)	6250478.5 (5601235.1–6947975.8)	165.2 (143.4 to 186.9)	112261.0 (105133.1–120,169)	98134.7 (87941.3–109085.6)	−12.6 (−19.8 to −5.4)
Netherlands	1411162.6 (1327405.6–1500714.6)	2001021.0 (1850004.7–2161649.5)	41.8 (38.0 to 45.4)	109815.3 (103297.4–116784.2)	85904.5 (79421.3–92800.3)	−21.8 (−23.8 to −19.8)
New Zealand	277736.9 (260494.7–296223.9)	431363.3 (395144.1–470330.6)	55.3 (51.1 to 59.2)	112255.6 (105286.7–119727.7)	83208.7 (76222.1–90725.4)	−25.9 (−27.9 to −24.0)
Norway	545430.0 (511679.0–582619.8)	539345.6 (495094.4–589737.6)	−1.1 (−3.7 to 1.3)	111636.2 (104728.2–119248.1)	82718.1 (75931.4–90446.6)	−25.9 (−27.8 to −24.1)
Poland	3328426.0 (3160318.5–3512520.6)	4275478.7 (3794588.2–4793821.3)	28.5 (16.5 to 40.9)	138128.4 (131152.0–145768.2)	97156.1 (86228.3–108934.9)	−29.7 (−36.2 to −22.8)
Portugal	1056917.2 (999025.9–1117863.4)	1462739.6 (1348230.6–1585297.7)	38.4 (34.2 to 42.5)	122492.9 (115783.5–129556.4)	85798.1 (79081.4–92986.8)	−30.0 (−32.1 to −27.9)
Slovak Republic	471257.6 (447987.3–495870.2)	580299.9 (497141.6–673575.2)	23.1 (7.3 to 40.7)	142580.1 (135539.7–150026.7)	103,182 (88395.8–119767.1)	−27.6 (−37.0 to −17.3)
Slovenia	177711.1 (151037.5–208942.1)	254566.5 (215771.1–298765.3)	43.2 (15.2 to 73.9)	130905.3 (111257.0–153910.6)	88632.7 (75125.3–104021.5)	−32.3 (−45.5 to −17.8)
Spain	3605739.0 (3380715.3–3850669.5)	5361371.6 (4921733.4–5839042.8)	48.7 (44.9 to 52.4)	104632.8 (98103.0–111740.3)	80906.1 (74271.7–88114.4)	−22.7 (−24.6 to −20.7)
Sweden	1141144.6 (1071043.8–1221133.6)	1244112.2 (1144042.9–1354071.3)	9.0 (6.5 to 11.4)	104444.0 (98027.9–111765.0)	80899.1 (74392.1–88049.3)	−22.5 (−24.3 to −20.9)
Switzerland	715638.6 (668188.3–767641.1)	903498.5 (823753.1–991460.8)	26.3 (22.7 to 29.8)	102513.8 (95716.7–109963.1)	75559.8 (68890.6–82916.1)	−26.3 (−28.4 to −24.2)
UK	7271204.4 (6868185.9–7718284.8)	7839162.3 (7246393.3–8483134.8)	7.8 (5.4 to 10.1)	117015.2 (110529.4–124210.0)	89296.9 (82544.6–96632.5)	−23.7 (−25.4 to −22.0)
USA	23113420.1 (21537371.3–24851044.2)	34444796.3 (31645372.0–37418073.0)	49.0 (46.6 to 51.1)	108304.3 (100919.3–116446.4)	95798.4 (88012.6–104067.7)	−11.5 (−13.0 to −10.3)

Table 2 shows YLD and YLL numbers and rates in 33 industrialised countries in 2019 and percent change from 1990 to 2019. As most countries showed increases in DALY numbers, YLD and YLL numbers also increased. However, percent changes were much greater in YLD numbers than in YLL numbers. Remarkably, in Norway where DALY numbers decreased from 1990 to 2019, YLD numbers increased by 35.1% while YLL numbers decreased by 13.3%. Regarding changes in YLL and YLD rates, decreases in DALY rates could be explained by the reduction in YLL rates since percent changes in YLD rates were lower than percent changes in YLL rates. For example, South Korea showed the highest reduction in DALY rates (Table 1). Percent changes in YLL rate was − 51.9% (95% UI −53.5 to −50.2), which was far higher than percent change in YLD rate (−4.4%, 95% UI −6.8 to −2.3).

**Table 2:** YLD and YLL numbers and rates per 100,000 people for adults 70 years and older in 33 industrialized countries between 1990 and 2019 and percent change from 1990 to 2019

Country	YLD (95% UI)				YLL (95% UI)			
	Numbers in 2019	Percent change (%)	Rates in 2019	Percent change (%)	Numbers in 2019	Percent change (%)	Rates in 2019	Percent change (%)
Australia	800327.6 (608604.1–1008490.1)	137.0 (132.0 to 141.9)	28595.6 (21745.3–36033.2)	2.4 (0.3 to 4.6)	1415770.3 (1391792.9–1,441,424)	49.0 (46.2 to 51.8)	50585.3 (49728.6–51501.9)	−35.6 (−36.8 to −34.4)
Austria	335218.8 (252910.6–428395.5)	61.4 (58.5 to 64.5)	27249.3 (20558.7–34823.5)	0.2 (−1.6 to 2.2)	694341.1 (683834.8–705509.5)	1.3 (−0.5 to 3.0)	56441.8 (55587.7–57349.6)	−37.1 (−38.2 to −36.0)
Belgium	437514.6 (331741.0–556282.5)	68.5 (65.5 to 71.7)	28,093 (21301.2–35719.1)	4.1 (2.2 to 6.0)	946445.5 (929346.0–964661.3)	10.3 (8.2 to 12.5)	60771.7 (59673.7–61941.3)	−31.9 (−33.2 to −30.5)
Canada	1246859.0 (946275.7–1565869.5)	129.9 (124.6 to 135.3)	27731.4 (21046.1–34826.5)	2.9 (0.5 to 5.2)	2339550.3 (2313913.4–2366460.9)	56.6 (54.8 to 58.6)	52034.0 (51463.8–52632.5)	−29.9 (−30.8 to −29.1)
Chile	383900.5 (288168.4–489,239)	182.0 (176.0 to 187.8)	26624.1 (19984.9–33929.4)	4.3 (2.1 to 6.5)	859559.1 (840579.3–879708.4)	78.3 (74.2 to 82.5)	59611.7 (58295.4–61009.1)	−34.0 (−35.5 to −32.5)
Czech Republic	423545.5 (319055.3–540318.6)	80.7 (76.3 to 85.1)	28878.5 (21754.1–36840.5)	−1.9 (−4.3 to 0.5)	998114.0 (851839.1–1167477.2)	−0.9 (−15.3 to 15.3)	68054.2 (58080.8–79601.9)	−46.2 (−54.0 to −37.4)
Denmark	212895.3 (161601.1–269377.9)	41.4 (38.2 to 44.4)	25942.1 (19691.7–32824.7)	−3.6 (−5.7 to −1.5)	494049.2 (481834.6–507046.2)	−2.7 (−5.2 to 0)	60201.8 (58713.4–61785.5)	−33.6 (−35.3 to −31.8)
Estonia	47985.6 (36186.5–61427.5)	52.7 (49.5 to 55.9)	26030.1 (19629.6–33321.8)	−1.7 (−3.8 to 0.3)	129752.4 (107585.4–154413.5)	−2.7 (−19.8 to 16.1)	70385.1 (58360.5–83762.7)	−37.4 (−48.4 to −25.3)
Finland	237078.2 (178074.6–303670.2)	92.2 (88.5 to 96.2)	26834.6 (20156.1–34372.1)	−0.6 (−2.5 to 1.5)	483931.6 (470270.6–498691.4)	19.0 (15.4 to 22.7)	54775.7 (53229.4–56446.3)	−38.5 (−40.3 to −36.5)
France	2411495.4 (1815627.3–3081988.2)	75.7 (71.8 to 79.9)	25544.0 (19232.2–32646.3)	−2.9 (−5.1 to −0.5)	4747533.5 (4675570.1–4823307.8)	19.4 (17.5 to 21.4)	50288.8 (49526.5–51091.4)	−34.0 (−35.0 to −32.9)
Germany	3679062.7 (2777284.8–4,655,304)	69.2 (66.0 to 72.4)	27746.9 (20945.8–35109.5)	3.1 (1.1 to 5.1)	8347243.8 (8226934.1–8491260.2)	10.4 (8.8 to 12.2)	62953.5 (62046.2–64039.7)	−32.7 (−33.7 to −31.6)
Greece	453860.5 (343180.3–570214.7)	85.2 (81.5 to 89.3)	26431.7 (19,986–33207.9)	2.4 (0.3 to 4.7)	1122899.2 (1101426.3–1146173.7)	39.6 (36.8 to 42.7)	65394.9 (64144.4–66750.3)	−22.8 (−24.3 to −21.1)
Hungary	365221.3 (276,247–464665.6)	45.5 (42.6 to 48.4)	27986.9 (21168.8–35607.3)	−5.6 (−7.4 to −3.7)	1047127.0 (902091.0–1212968.3)	2.8 (−11.7 to 18.9)	80241.3 (69127.2–92949.7)	−33.3 (−42.7 to −22.8)
Ireland	127399.7 (96499.8–160819.5)	84.6 (81.2 to 88.4)	26418.7 (20011.0–33348.9)	2.9 (0.9 to 4.9)	276691.3 (268627.7–285364.5)	1.1 (−2.1 to 4.3)	57,377 (55704.9–59175.6)	−43.7 (−45.5 to −41.9)
Israel	188767.2 (142342.9–239556.9)	150.2 (145.8 to 155.2)	25198.4 (19001.2–31978.3)	1.8 (−0.1 to 3.8)	393648.1 (385245.4–402727.0)	62.3 (58.2 to 66.4)	52547.8 (51426.1–53759.7)	−34 (−35.7 to −32.4)
Italy	2868166.1 (2164905.6–3654619.3)	81.8 (79.5 to 84.0)	27869.4 (21035.9–35511.2)	−2.2 (−3.4 to −1.0)	5644994.8 (5595190.0–5,692,949)	27.3 (26.1 to 28.4)	54851.2 (54367.3–55317.2)	−31.5 (−32.1 to −30.9)
Japan	6999940.3 (5,288,503–8909147.7)	176.5 (171.9 to 181.5)	25391.9 (19183.7–32317.4)	−0.9 (−2.5 to 0.9)	12271500.5 (12155959.1–12388599.7)	87.2 (85.4 to 89.2)	44514.1 (44,095–44938.9)	−32.9 (−33.5 to −32.2)
South Korea	74683.8 (56401.5–95,311)	295.2 (285.4 to 304.0)	26547.3 (20048.6–33879.5)	−4.4 (−6.8 to −2.3)	2596875.1 (2511864.7–2685850.9)	98.9 (92.2 to 105.9)	79239.7 (69,579–91519.9)	−51.9 (−53.5 to −50.2)
Latvia	106756.9 (80,686–136080.5)	30.2 (27.7 to 32.4)	26681.7 (20165.8–34010.5)	−3.8 (−5.6 to −2.1)	222920.0 (195742.1−257467.1)	−2.2 (−14.4 to 13.4)	75700.7 (65140.4–87260.9)	−27.7 (−36.7 to −16.1)
Lithuania	1780685.2 (1343921.7–2263409.1)	52.9 (50.2 to 55.5)	27957.4 (21100.0–35536.3)	−2.6 (−4.3 to −0.9)	302888.4 (260635.2–349142.2)	19.8 (2.8 to 38.7)	70177.3 (62430.8–78104.4)	−23.7 (−34.5 to −11.6)
Mexico	621841.7 (474634.6–779924.3)	189.9 (186.2 to 193.6)	26695.9 (20376.2–33482.4)	−4.5 (−5.7 to −3.2)	4469793.3 (3976396.0–4974694.1)	156.6 (127.8 to 186.0)	59208.6 (57976.4–60,527)	−15.4 (−24.9 to −5.7)
Netherlands	145660.5 (110195.1–185346.9)	87.0 (82.8 to 91.7)	28097.5 (21256.3–35752.9)	3.2 (0.8 to 5.8)	1379179.4 (1350475.7–1409888.7)	27.9 (25.1 to 30.8)	55111.2 (54271.9–55949.6)	−29.5 (−31 to −27.8)
New Zealand	185580.9 (140426.2–236130.6)	108.7 (105.2 to 111.8)	28462.1 (21536.8–36214.8)	−0.4 (−2.1 to 1.1)	285702.8 (281351.7–290049.2)	37.4 (35.0 to 39.9)	54256.0 (53411.6–55117.4)	−34.4 (−35.6 to −33.2)
Norway	1182606.5 (896,504–1511103.8)	35.1 (33.4 to 36.9)	26873.6 (20372.2–34338.4)	1.2 (0–2.6)	353764.7 (348259.0–359380.9)	−13.3 (−14.9 to −11.7)	70282.5 (61210.6–79714.9)	−35.0 (−36.2 to −33.8)
Poland	457981.5 (345674.9–581132.6)	75 (72.6 to 77.2)	26863.2 (20275.8–34086.8)	−4.2 (−5.5 to −3.0)	3092872.2 (2693652.9–3507958.6)	16.6 (1.7 to 32.1)	58934.8 (57767.9–60176.8)	−36.2 (−44.3 to −27.7)
Portugal	1327650.0 (997,793–1683477.3)	95.9 (91.5 to 100.1)	25275.3 (18995.6–32049.4)	−0.8 (−3.1 to 1.3)	1004758.1 (984864.0–1025932.7)	22.1 (19.5 to 24.8)	49438.3 (47819.9–51132.2)	−38.2 (−39.5 to −36.8)
Slovak Republic	150165.8 (113606.5–192046.9)	66.8 (63.4 to 70.0)	26700.7 (20200.1–34147.5)	−2 (−4.0 to −0.1)	430134.1 (357330.1–514115.2)	12.8 (−6.0 to 34.4)	76481.3 (63536.2–91413.8)	−33.7 (−44.8 to −21.0)
Slovenia	81413.8 (61462.1–103835.8)	105.4 (101.4 to 109)	28345.9 (21399.3–36152.6)	−2.9 (−4.8 to 1.2)	173152.7 (141878.4–213360.0)	25.4 (−6.6 to 63.4)	60286.7 (49398.0–74285.8)	−40.7 (−55.8 to −22.8)
Spain	1788970.4 (1351853.3–2259792.7)	94 (89.6 to 98.8)	26996.5 (20400.2–34101.5)	0.9 (−1.4 to 3.4)	3572401.2 (3516725.0–3631803.4)	33.1 (31.1 to 35.4)	53909.5 (53069.3–54805.9)	−30.8 (−31.8 to −29.6)
Sweden	408366.9 (309393.4–519287.3)	44.2 (42.1 to 46.5)	26554.3 (20118.5–33767.0)	2.4 (0.9 to 4.1)	835745.3 (826132.5–846262.8)	−2.6 (−3.9 to −1.1)	54344.8 (53719.8–55028.7)	−30.8 (−31.7 to −29.7)
Switzerland	317696.8 (240207.2–406,063)	65.5 (62.2 to 68.7)	26569.0 (20088.6–33959.1)	−3.4 (−5.3 to −1.5)	585801.8 (573880.0–598605.3)	11.9 (9.4 to 14.4)	48990.7 (47993.7–50061.5)	−34.7 (−36.1 to −33.2)
UK	2425735.7 (1836344.8–3091298.9)	47.0 (45.2 to 48.9)	27631.9 (20918.0–35213.4)	4.1 (2.8 to 5.4)	5413426.6 (5369819.5–5459659.3)	−3.7 (−4.5 to −2.8)	61665.1 (61168.3–62191.7)	−31.8 (−32.4 to −31.2)
USA	12223406.7 (9451188.3–15217980.1)	81.4 (78.4 to 84.7)	33995.9 (26285.8–42324.5)	7.7 (5.9 to 9.7)	22221389.7 (22063859.1–22382036.8)	35.7 (34.7 to 36.7)	61802.4 (61364.3–62249.2)	−19.5 (−20.0 to −18.9)

**Fig. 3:**
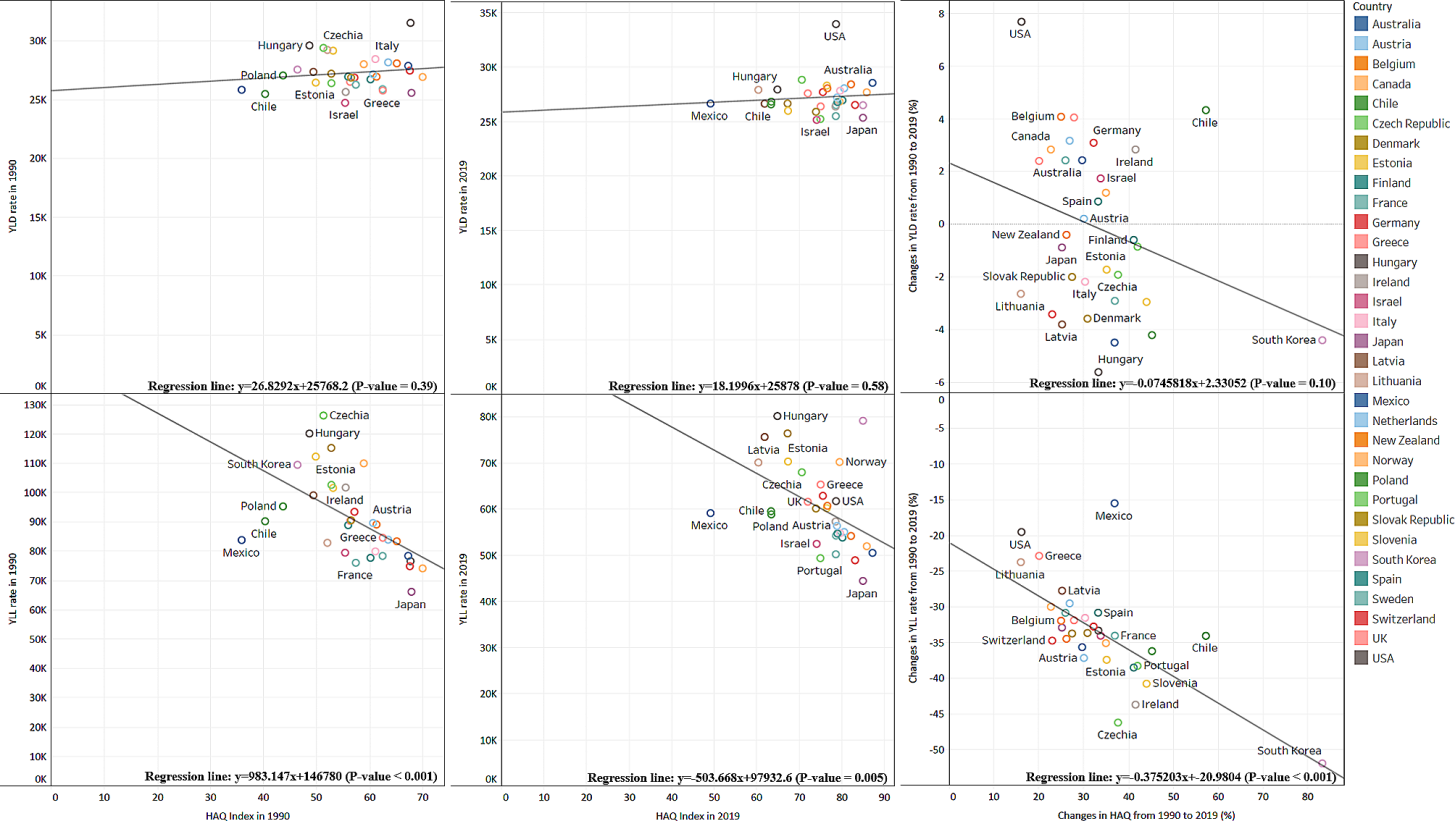
Association between YLD and YLL rates for those aged over 70 and HAQ Index from 1990 to 2019

Figure 3 shows association between YLD and YLL for those over 70 years and HAQ in 1990 and 2019. There were significant associations between YLL and HAQ in 1990 and 2019. However, associations between YLD and HAQ were not statistically significant. Similarly, increases in HAQ were significantly associated with decreases in YLL (p-value < 0.001), whereas changes in HAQ were not associated with changes in YLD (p-value = 0.10).

## Discussion

### Main findings

In this study, we observed a marked increase in life expectancy and HALE among individuals aged 70–74 years in 33 industrialised countries. These increases were strongly related to the HAQ index between 1990 and 2019. However, we observed an increase in unhealthy years of life alongside an increase in HAQ index during the same period. There was an overall reduction in DALY rate and the decreases in YLL rates were more pronounced compared to that in YLD rates. However, there were increases in DALY numbers, among which increases of YLD were more pronounced than those of YLL numbers between 1990 and 2019. Regarding the association between YLD or YLL rates and HAQ index, we found that the YLL rate, but not YLD, was related to HAQ index in 1990 and 2019.

### Interpretation and policy implications

Our findings showed an increase in life expectancy and HALE among individuals aged 70–74 years across all industrialized countries over the three decades. This is in affirmation of a previous study [3]. As socioeconomic development and promoting health behaviour are related to mortality and life expectancy, these may also account for changing life expectancy and HALE [29–32]. Furthermore, another possible explanation for increases of both life expectancy and HALE might be related to improvement in performances of healthcare systems because our findings showed enhanced healthcare performances for older people across the last three decades [15] and significant associations between life expectancy or HALE and HAQ in both 1990 and 2019, as well as between changes in life expectancy or HALE and changes in the HAQ index.

Although HALE for individuals aged 70–74 years increased with the improvement of life expectancy, unhealthy years of life also increased during the study period despite an increase in the HAQ index. Progress in medical care might explain the declining premature mortality and increased survival among weak older people [12], potentially leading to an extended period of living with poor health. Our findings demonstrated that decreases in DALY rates were mainly attributed to reductions in YLL (premature mortality burden). However, patterns of YLD rates were stable or increased in some countries. Notably, YLD numbers substantially increased in almost all countries. These changes in YLD were largely driven by an increased prevalence rate for chronic diseases and an ageing population [33].

Our findings showed strong evidence of associations between YLL rates for individuals aged over 70 years and HAQ index for the post-working population between 1990 and 2019, but no statistical evidence of association between YLD and HAQ. Over the three decades, the HAQ might have been associated with improved healthcare performance in acute care. Particularly, South Korea showed the most significant rises in life expectancy and HALE due to improvements of healthcare system performance. However, this was coupled with considerable increases in unhealthy years of life. According to OECD Health Statistics, healthcare utilisation and access in South Korea ranked highest among OECD countries [34]. This extensive healthcare utilisation and access may contribute to reduced mortality rates. However, some indicators reflecting the quality of primary care, such as hospital admission rates of chronic conditions like hypertension or diabetes, were below than the OECD average [34]. Moreover, healthcare expenses for chronic disease constituted 85.0% of the total medical costs [35]. It is imperative to focus more on further reducing morbidity or disability caused by chronic disease.

As high-income countries have experienced fast population ageing in recent years, healthcare systems are poised to face several challenges. With the burden of diseases likely to persist and additional years spent in poor health increasing, healthcare needs and expenditures substantially increase [36]. This suggests a considerable healthcare burden and also great economic and social-related burden [37, 38]. Therefore, an age-friendly healthcare system needs to be reorganised and modified better to meet needs of the increasing number of older adults [33].

### Strengths and limitations of this study

This research has some strengths. By analysing data from 33 industrialised countries, it provides evidence regarding changes in life expectancy and HALE for older people, and their association with healthcare access and quality. Furthermore, it highlights the association between differences in HAQ for the post-working population and burden of diseases indicated by YLL and YLD between 1990 and 2019.

This study also has several limitations. Since this study used data from the GBD 2019, it had general limitations of the GBD 2019 as described previously [39]. One such limitation was a variation in data quality, where the GBD 2019 gathered primary data from registration, vital statistics, and survey for each age and country. However, estimates for certain age groups and countries lacking sufficient primary data heavily relied on modelling, resulting in considerable uncertainty intervals. Secondly, prior research highlighted limitations of the HAQ Index [15]. In particular, it cannot separate specific characteristics of healthcare access or quality, such as differentiating quality from other care or estimating the influence of acceptability or cultural barriers [15], suggesting the HAQ may not capture healthcare system in some areas. For example, an increased disease burden may be related to greater access to healthcare among older people. However, if well controlled, it can have a relatively small impact on quality of life. Further investigation would be necessary, considering factors that the HAQ may not capture. Thirdly, fluctuations in life expectancy, HALE, and disease burden over the three decades might have occurred. However, our analysis was based on data from only two time points, which limited our investigation into comprehensive changes in life expectancy, HALE, and burden of diseases for individuals aged over 70 years. Last, our study did not cover a different range of other factors contributing to healthy aging, such as environments, life-course factors, and well-being [40], and various causes of disease, although the GBD 2019 provided estimates for some risk factors and causes of disease that could be potentially associated with disease burden. Particularly, an age-friendly social and community environment, including broader social services, systems and policies, socioeconomic development, as well as social relationships, may play an important role in promoting healthy ageing [40], suggesting that these factors may account for differences in good health status of older adults across countries.

To achieve healthy aging, a concentrated effort may be required to address the research and knowledge gaps. For example, the World Health Organization reported a plan for the Decade of Healthy Ageing (2021–2030), which includes four areas of action: age-friendly environments, combating ageism, integrated care, and long-term care [41]. Thus, further investigation is needed to better understand the association between disease burden and the healthcare system while accounting for those factors.

## Conclusions

Life expectancy and HALE showed an upward trend across all industrialised countries over the three decades, indicating improved performance of healthcare systems. However, despite these gains, there was an increase in unhealthy years of life, suggesting a sustained morbidity burden among the ageing population. This highlights the potential necessity of enhancing the healthcare system to effectively address the healthcare burden for the ageing population. We might need to develop and implement targeted public health strategies for alleviating disability and improving functional ability with a flexible ageing-friendly healthcare system.

### Electronic supplementary material

Below is the link to the electronic supplementary material.

Supplementary Material 1

## Data Availability

The datasets generated and/or analysed during the current study are available in the Global Health Data Exchange (GHDx), [https://ghdx.healthdata.org].
