# Supplementary material for: Living longer but in poor health: healthcare system responses to ageing populations in industrialised countries based on the Findings from the Global Burden of Disease Study 2019
Source: BMC Public Health. 2024 Feb 22;24:576. doi: 10.1186/s12889-024-18049-0 (PMC10885395; doi:10.1186/s12889-024-18049-0)
Supplement: Supplementary file 1 — Supplementary Material 1 [file 12889_2024_18049_MOESM1_ESM.docx]

Supplementary Table 1. Life expectancy, HALE, and unhealthy years at 70-74 years of life in 1990 and 2019

| **Country** | **Life expectancy in 1990** | **Life expectancy in 2019** | **HALE in 1990** | **HALE in 2019** | **Unhealthy years of life in 1990** | **Unhealthy years of life in 2019** |
| --- | --- | --- | --- | --- | --- | --- |
|  | **Years** | **Years** | **Years** | **Years** | **Years^a^ (%)**^b^ | **Years^a^ (%)**^b^ |
| Australia | 13.8 | 17.2 | 9.9 | 12.1 | 4(28.8) | 5.1(29.8) |
| Austria | 13.2 | 16.6 | 9.6 | 12 | 3.6(27.1) | 4.6(27.8) |
| Belgium | 13.2 | 16.2 | 9.6 | 11.6 | 3.6(27) | 4.6(28.5) |
| Canada | 14.3 | 17.1 | 10.3 | 12.1 | 4(27.9) | 5(29.1) |
| Chile | 12.8 | 15.9 | 9.5 | 11.5 | 3.3(26.1) | 4.4(27.6) |
| Czech Republic | 10.8 | 14.8 | 7.7 | 10.4 | 3.1(28.9) | 4.4(29.6) |
| Denmark | 13 | 15.8 | 9.5 | 11.6 | 3.5(27.2) | 4.3(26.9) |
| Estonia | 11.5 | 15 | 8.5 | 11.1 | 3(26.3) | 3.9(26.1) |
| Finland | 13.1 | 16.6 | 9.5 | 12 | 3.6(27.4) | 4.6(27.8) |
| France | 14.4 | 17.7 | 10.6 | 13.1 | 3.8(26.3) | 4.6(26.2) |
| Germany | 13 | 15.9 | 9.5 | 11.5 | 3.5(26.8) | 4.5(28) |
| Greece | 13.3 | 15.8 | 9.8 | 11.6 | 3.5(26.2) | 4.2(26.5) |
| Hungary | 11.1 | 13.9 | 7.8 | 10 | 3.2(29.4) | 3.9(28.3) |
| Ireland | 12 | 16.1 | 8.9 | 11.7 | 3.1(26) | 4.4(27.3) |
| Israel | 13.6 | 16.9 | 10.2 | 12.5 | 3.5(25.4) | 4.4(26.1) |
| Italy | 13.8 | 16.9 | 9.8 | 12.1 | 4(29) | 4.8(28.4) |
| Japan | 14.9 | 18.6 | 10.9 | 13.7 | 4(26.6) | 4.9(26.4) |
| Korea | 11.7 | 17 | 8.5 | 12.4 | 3.2(27.2) | 4.5(26.7) |
| Latvia | 11.8 | 14.2 | 8.6 | 10.4 | 3.2(27.4) | 3.8(26.5) |
| Lithuania | 12.5 | 14.5 | 9.1 | 10.7 | 3.4(27.3) | 3.9(26.6) |
| Mexico | 13.1 | 14.9 | 9.2 | 10.6 | 3.9(29.7) | 4.3(28.7) |
| Netherlands | 13.5 | 16 | 10 | 11.5 | 3.6(26.3) | 4.4(27.7) |
| New Zealand | 13.4 | 16.5 | 9.5 | 11.7 | 3.9(29) | 4.9(29.3) |
| Norway | 13.6 | 16.7 | 9.7 | 11.8 | 3.9(28.6) | 5(29.6) |
| Poland | 11.7 | 14.9 | 8.4 | 10.9 | 3.3(28) | 4.1(27.3) |
| Portugal | 12.4 | 16.3 | 9 | 11.9 | 3.4(27.4) | 4.5(27.3) |
| Slovak Republic | 11.4 | 14.1 | 8.3 | 10.3 | 3.1(27.1) | 3.8(27.1) |
| Slovenia | 12.4 | 16.3 | 8.8 | 11.6 | 3.6(28.8) | 4.7(28.8) |
| Spain | 13.9 | 17.2 | 10.1 | 12.5 | 3.8(27.3) | 4.7(27.4) |
| Sweden | 14 | 16.7 | 10.3 | 12.1 | 3.7(26.4) | 4.6(27.5) |
| Switzerland | 14.4 | 17.6 | 10.4 | 12.8 | 4(27.9) | 4.8(27.5) |
| UK | 13 | 15.8 | 9.5 | 11.3 | 3.5(26.8) | 4.5(28.2) |
| USA | 14.3 | 15.9 | 9.7 | 10.4 | 4.6(32.3) | 5.6(34.9) |

^a^ Difference between life expectancy and HALE (life expectancy – HALE)

^b^ Proportion of unhealthy years of life [(life expectancy -HALE)/ life expectancy]
